# Embedding Task-Based Neural Models into a Connectome-Based Model of the Cerebral Cortex

**DOI:** 10.3389/fninf.2016.00032

**Published:** 2016-08-03

**Authors:** Antonio Ulloa, Barry Horwitz

**Affiliations:** ^1^Section on Brain Imaging and Modeling, National Institute on Deafness and Other Communication Disorders, National Institutes of HealthBethesda, MD, USA; ^2^Neural Bytes LLCWashington, DC, USA

**Keywords:** computational modeling, neural networks, visual object processing, The Virtual Brain, fMRI, human, brain

## Abstract

A number of recent efforts have used large-scale, biologically realistic, neural models to help understand the neural basis for the patterns of activity observed in both resting state and task-related functional neural imaging data. An example of the former is The Virtual Brain (TVB) software platform, which allows one to apply large-scale neural modeling in a whole brain framework. TVB provides a set of structural connectomes of the human cerebral cortex, a collection of neural processing units for each connectome node, and various forward models that can convert simulated neural activity into a variety of functional brain imaging signals. In this paper, we demonstrate how to embed a previously or newly constructed task-based large-scale neural model into the TVB platform. We tested our method on a previously constructed large-scale neural model (LSNM) of visual object processing that consisted of interconnected neural populations that represent, primary and secondary visual, inferotemporal, and prefrontal cortex. Some neural elements in the original model were “non-task-specific” (NS) neurons that served as noise generators to “task-specific” neurons that processed shapes during a delayed match-to-sample (DMS) task. We replaced the NS neurons with an anatomical TVB connectome model of the cerebral cortex comprising 998 regions of interest interconnected by white matter fiber tract weights. We embedded our LSNM of visual object processing into corresponding nodes within the TVB connectome. Reciprocal connections between TVB nodes and our task-based modules were included in this framework. We ran visual object processing simulations and showed that the TVB simulator successfully replaced the noise generation originally provided by NS neurons; i.e., the DMS tasks performed with the hybrid LSNM/TVB simulator generated equivalent neural and fMRI activity to that of the original task-based models. Additionally, we found partial agreement between the functional connectivities using the hybrid LSNM/TVB model and the original LSNM. Our framework thus presents a way to embed task-based neural models into the TVB platform, enabling a better comparison between empirical and computational data, which in turn can lead to a better understanding of how interacting neural populations give rise to human cognitive behaviors.

## Introduction

Large-scale neural network models aim to shed light on the mechanisms used by the brain to accomplish goal-directed behavioral tasks. Often, for computational efficiency large-scale neural models (LSNM) comprise the minimum number of brain regions that are necessary to simulate a given task. However, by restricting the number of brain regions to only a few, neural network models may miss the contributions and constraints that the “rest of the brain” could provide on both the simulated task and the sought-after mechanisms.

In the past few years, there has been increased interest in using LSNM with functional neuroimaging data such as PET, fMRI, EEG/MEG in order to help understand the neural basis for the patterns of activity observed in the imaging data. Examples can be found for resting state data (Honey et al., 2007; Alstott et al., 2009; Cabral et al., 2011, 2012; van Dellen et al., 2013), and for task-based data (Tagamets and Horwitz, 1998; Horwitz and Tagamets, 1999; Corchs and Deco, 2002; Deco et al., 2004; Husain et al., 2004; Horwitz et al., 2005; Robinson et al., 2005; Ulloa et al., 2008; Peters et al., 2010; Bojak et al., 2011; Banerjee et al., 2012a; Furtinger et al., 2014). Such a modeling framework requires three submodels: a structural model of the anatomical links between brain regions that provides the interregional connection weights; one or more neural models at each node for generating the neural activity; and a forward model that transforms a combination of the neural activity into a neuroimaging signal.

Recently, a software platform called The Virtual Brain (TVB) has become available that facilitates applying LSNM to neuroimaging data in a whole brain framework (Jirsa et al., 2010; Ritter et al., 2013; Sanz Leon et al., 2013, 2015). The platform consists of several structural models (e.g., monkey CoCoMac data Kotter, 2004; human diffusion spectrum imaging (DSI) connectome Hagmann et al., 2008), several neural models that can represent the activity of a node (e.g., Wilson-Cowan unit Wilson and Cowan, 1972; Jansen-Rit unit Jansen and Rit, 1995), and several forward models that can convert some measure of neural activity into either fMRI (e.g., Friston et al., 2000) or EEG/MEG signals (e.g., Sarvas, 1987). A recent development incorporates an automated pipeline for constructing personalized virtual brains from multimodal neuroimaging data, including individual diffusion-weighted MRI data (Schirner et al., 2015).

To date, most of the applications of TVB (and similar LSNM that employ connectome-type structural models) have been utilized to simulate resting state data or simple stimulation studies using neural noise propagating throughout the brain (e.g., Jirsa et al., 2010; Cabral et al., 2011; Ritter et al., 2013; Ponce-Alvarez et al., 2015). However, as noted above, there exists a number of LSNM that simulate specific cognitive tasks, and some of these models have been employed to simulate human functional neuroimaging data. One important difference between these two categories of neural models is in the nature of the anatomical submodel that each type employs. In general, the weights of the anatomical interregional connections found in the resting state simulations are based on diffusion tensor/spectrum imaging data (e.g., Hagmann et al., 2008). For the task-based simulations, a more detailed set of anatomical connection weights is needed, since it is usually by means of the strength and specific organization of these interregional connections that the cognitive task can be implemented (we will provide a specific example in the next section).

Thus, the question arises as to how to modify a structural model like the ones provided by TVB to implement the specific anatomical assumptions constituting the hypotheses underlying a specific cognitive task. In this paper, we demonstrate how to do this by embedding one of two models that our laboratory has previously developed (Tagamets and Horwitz, 1998; Horwitz and Tagamets, 1999; Husain et al., 2004; Horwitz et al., 2005) into TVB architecture. These models perform a short-term memory task for either visual objects (Tagamets and Horwitz, 1998) or auditory objects (Husain et al., 2004). Simulated neuronal data from both models were shown to generally agree with neurophysiological recordings from non-human primates and simulated functional neuroimaging data matched human PET and fMRI empirical data. The model used in this paper will be the visual model.

Below, we briefly describe our in-house visual object short-term memory model as well as TVB simulator. Next, we describe the steps we followed to embed the LSNM modules into the nodes of one of the structural models contained in TVB—the human connectome model of Hagmann et al. (2008). We then present results of simulated neuronal activity, simulated fMRI BOLD signal and simulated functional connectivity from the enhanced-TVB model and compare them with that of the stand-alone version of the task-based LSNM. A good match between the stand-alone and enhanced-TVB simulated data sets will demonstrate that we have successfully combined the LSNM and TVB models. Finally, we discuss our results and give directions for future work.

## Overview: visual object processing model and the virtual brain

### Visual object processing model

**Figure 1:**
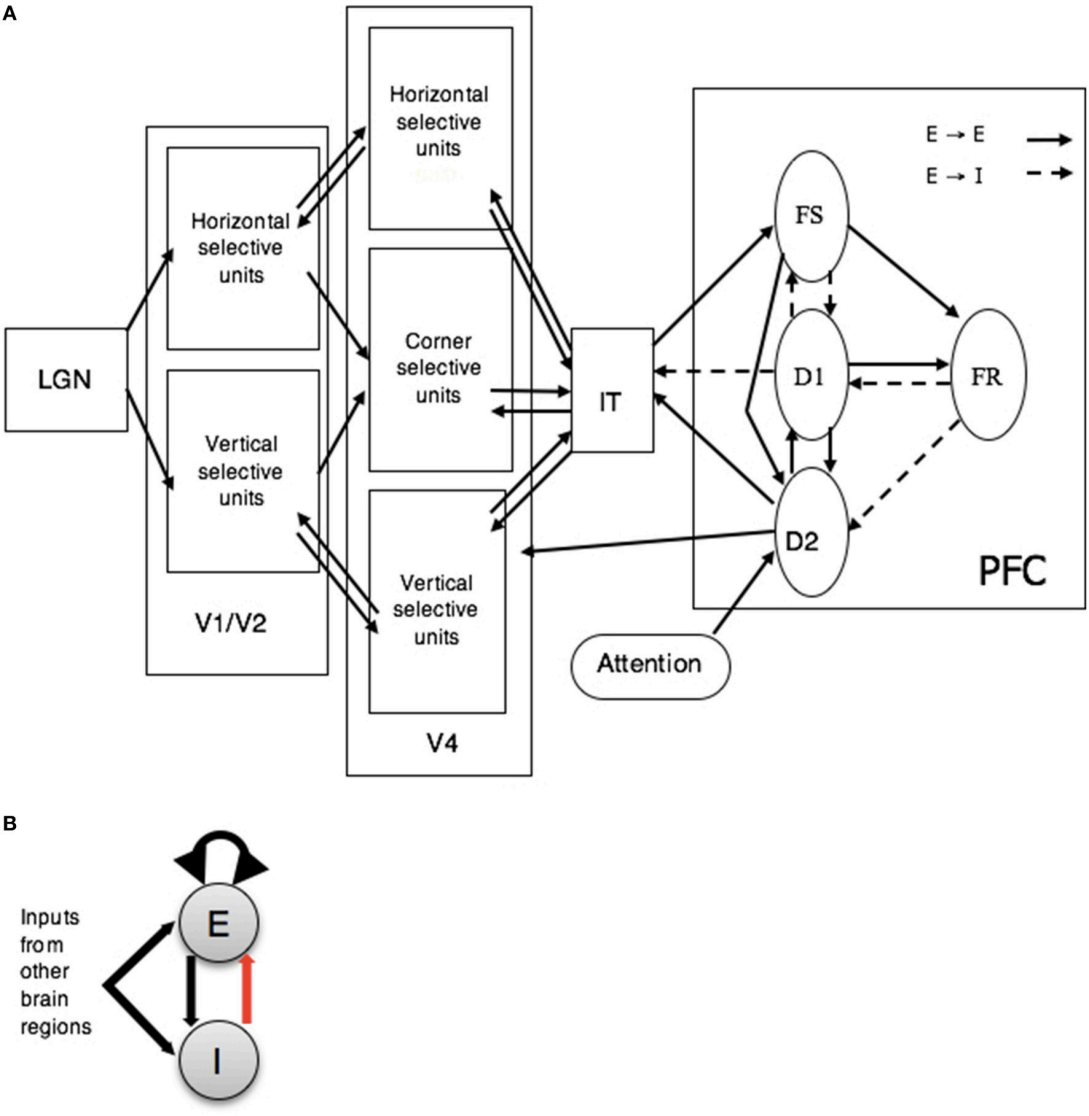
**(A)** Large-scale neural models of visual short-term memory. FS, D1, D2, and FR represent distinct neuronal populations within PFC (see Tagamets and Horwitz, 1998 for details). Abbreviations: LGN, lateral geniculate nucleus; V1–V2, primary and secondary visual cortex; V4, extrastriate visual cortex; IT, inferior-temporal cortex; PFC, prefrontal cortex. **(B)** Basic neuronal population unit (Wilson-Cowan) of the visual model of short term memory. Each unit has an excitatory (E) and an inhibitory (I) element. Dark arrows represent excitation and the red arrow represents inhibition. Adapted from Horwitz et al. (2005).

Our in-house visual (Tagamets and Horwitz, 1998) object processing model consists of interconnected neuronal populations representing the cortical ventral pathway that has been shown to process primarily the features of a visual object. This stream begins in striate visual cortex, extends into the inferior temporal lobe and projects into ventrolateral prefrontal cortex (Ungerleider and Mishkin, 1982; Haxby et al., 1991; McIntosh et al., 1994). The regions that comprise the visual model include ones representing primary and secondary visual cortex (V1/V2), area V4, anterior inferotemporal cortex (IT), and prefrontal cortex (PFC) (see Figure 1A). Each of these regions contain one or more neural populations with different functional attributes (see caption to Figure 1 for details). This model was designed to perform a short-term recognition memory delayed match-to-sample (DMS) task during each trial of which a stimulus S1 is presented for a certain amount of time, followed by a delay period in which S1 has to be kept in short-term memory. When a second stimulus (S2) is presented, the model has to respond as to whether S2 matches S1. The model can also perform a control task, passive perception of the stimuli, in which no response is required. Multiple trials of the active and passive tasks constitute a simulated functional neuroimaging study.

The key feature used to define a visual object was shape. Model neurons in V1 and V4 were assumed to be orientation selective (for simplicity, horizontal and vertical orientations were used). The structural submodels employed were based on known monkey neuroanatomical data. An important assumption for the visual model, inferred from such experimental data, was that the spatial receptive field on neurons increased along the ventral processing pathway (see Tagamets and Horwitz, 1998 for details). As a result, the model neurons in V4 responded to stimuli that had longer horizontal and vertical lines than did model neurons in the V1. Furthermore, because of the increasing receptive field size, the simulated image presented to the model was represented in the model IT as a distributed representation.

An important aspect of the LSNM was how the model maintained a representation of the S1 during the delay period of a trial and decided if the S2 stimulus matched the S1 stimulus. This was accomplished by the arrangement and strengths of connections between the four PFC subpopulations, along with their connections back to the IT module. The four types of simulated neurons in the model PFC were based on experimental findings of Funahashi et al. (1990) who found neurons in monkey PFC with four different response firing patterns during a delayed response task. Also of importance in our model is how the task instructions are provided to the model. Our LSNM performs two tasks, the DMS task and a control task (passive viewing of degraded shapes) in which stimuli are presented, but nothing is maintained in short-term memory; the task that is performed is controlled by the setting of an input (from outside the model) to one of the PFC neural populations (labeled D2 in Figure 1). Although, this control variable is labeled as “attention” in Figure 1, it embodies a number of top-down processes including attending to the stimuli. If the second stimulus of the trial, S2, matches the first, S1, then the activity in the FR submodule rises above a threshold value, and that constitutes a positive response.

Each neuronal population consisted of 81 microcircuits, each representing a cortical column. The model employed modified Wilson-Cowan units (an interacting excitatory and inhibitory pair of elements for which spike rate was the measure of output neural activity) as the microcircuit (see Figure 1B; Wilson and Cowan, 1972; Tagamets and Horwitz, 1998). The input synaptic activity to each neuronal unit can also be evaluated and combinations of this activity were related to the fMRI or MEG/EEG signals via a forward model. In this paper, we will only consider simulated fMRI (Horwitz and Tagamets, 1999).

Half the neural populations within the stand-alone model were “non-task-specific” neurons (Horwitz et al., 2005) that served as noise generators to “task-specific” neurons that processed shapes during the DMS task. The model generated time series of simulated electrical neuronal and synaptic activity for each module that represents a brain region. The time series of synaptic activity, convolved with a hemodynamic response function, was then used to compute simulated fMRI BOLD signal for each module representing a brain region, as well as functional connectivity among key brain regions (see Horwitz et al., 2005 for details on this method). The model is able to perform the DMS task, generate simulated neural activities in the various brain regions that matches empirical data from non-human preparations, and produces simulated functional neuroimaging data that generally agree with human experimental findings (see Tagamets and Horwitz, 1998; Horwitz et al., 2005 for details). In the current paper, we replace the non-task-specific neurons by noise-generated activity from neural elements in TVB.

### The virtual brain

The Virtual Brain (TVB) software (Sanz Leon et al., 2013, 2015) is a simulator of primarily resting state brain activity that combines: (i) white matter structural connections among brain regions to simulate long-range connections, and (ii) a given neuronal population model to simulate local brain activity. It also employs forward models that convert simulated neural activity into simulated functional neuroimaging data. TVB source code and documentation are freely available from https://github.com/the-virtual-brain.

In the current paper, for the structural model, we have chosen to use the DSI-based connectome described by Hagmann et al. (2008), which contains 998 nodes and 66 brain regions. For the neural model for each node, we have chosen to use Wilson-Cowan population neuronal units (Wilson and Cowan, 1972) to model the local brain activity because our in-house LSNM simulators used modified Wilson-Cowan equations as their basic neuronal unit (the two types of Wilson-Cowan units differ primarily in that they have different weights between and within unit elements, and the modified Wilson-Cowan units employed in the LSNM nodes have no inhibitory-to-inhibitory self-connection). Our forward model that converts simulated neural activity into simulated fMRI is a modification of the Balloon-Winkessel model of Friston et al. (2000) and Stephan et al. (2007) that is included in the TVB.

## Methods

In the following, we describe the way in which we embedded the LSNM visual model of Tagamets and Horwitz (1998) and Horwitz et al. (2005) into the TVB. Within the LSNM, connections and parameter choices closely follow those in the original papers. Likewise, the connections and parameter choices among TVB nodes closely follow those described by Sanz Leon et al. (2015).

### Task-based model node placement in the TVB

The connectome derived by Hagmann et al. (2008) serves as a source of neural noise to our task-based neural model. Such a connectome was obtained by averaging the weighted network of five experimental subjects, where each one of the 998 nodes represents a region of interest (ROI) covering a surface area of approximately 1.5 cm^2^. The connection weights among the nodes represent cortico-cortical connections given by white matter connection density among the given nodes. As stated above, each node is represented by a Wilson-Cowan population unit and thus each node is assumed to be comprised of one excitatory and one inhibitory neural population. We implemented noise as an additive term to the stochastic Euler integration scheme provided by the TVB software.

**Figure 2:**
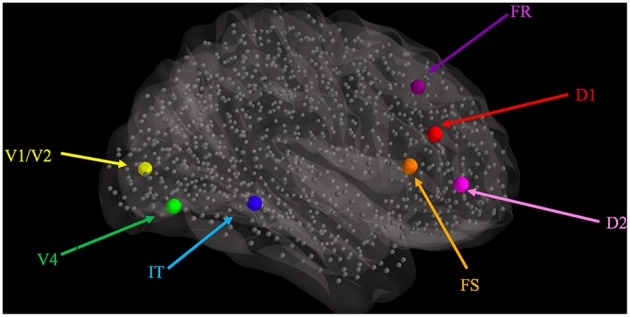
**Graphical representation of the locations where visual LSNM modules were embedded, relative to the location of the 998 nodes (smaller gray spheres) comprising the structural connectome of Hagmann et al. (2008), as represented in TVB**. The larger spheres represent the location of the visual processing LSNM modules, as indicated with colored labels. Connections among LSNM modules and among nodes are not shown but were preserved upon embedding.

**Figure 3:**
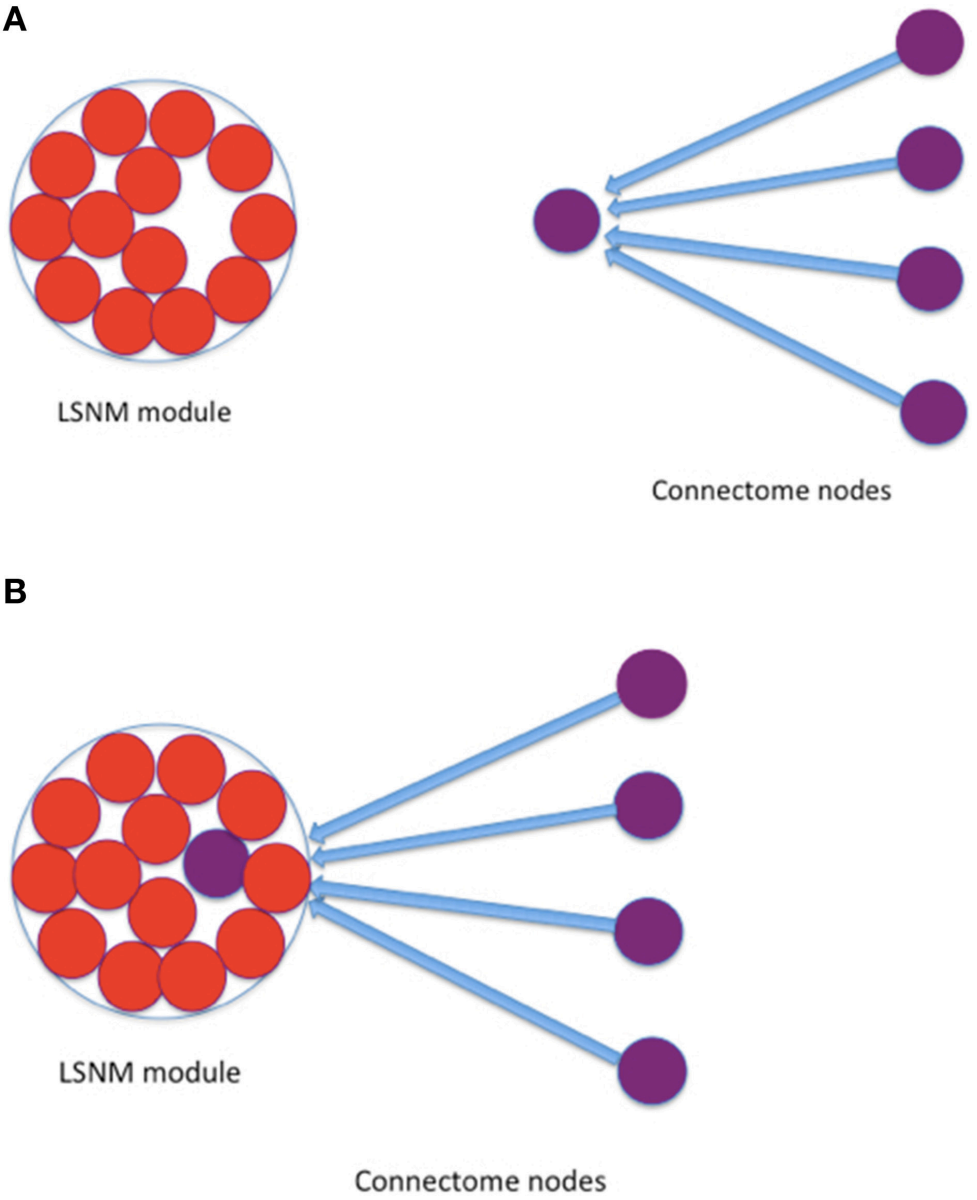
**Graphical depiction that shows how a given LSNM module was embedded into a TVB node**. **(A)** An LSNM module is composed of several neuronal population units (red circles) and we find the TVB node (purple circle with incoming connections) that is closest to the hypothesized Talairach location of the LSNM module. Several other nodes in TVB have connections to the designated “host” node (blue arrows); **(B)** we embed the LSNM module by connecting all TVB nodes that have connections to the host TVB node to the embedded LSNM units.

To facilitate the interaction, at a computational level, between our in-house LSNM simulator and the TVB source code (written in Python), we ported our simulator to the Python language. We then embedded, separately, our model of visual object processing into corresponding nodes within the TVB model (source code can be found at https://github.com/NIDCD/lsnm_in_python). Four steps were followed to embed each of the LSNM submodules of our model into the connectome:

1. We identified Talairach coordinates (Talairach and Tournoux, 1988) for each of the modules in the LSNM visual model (see Table 1) in the visual experimental literature, primarily from Haxby et al. (1995).
2. We used those hypothesized Talairach coordinates to find the closest connectome node (also shown in Table 1) to each LSNM module's location and thus designated “host” TVB nodes for each of the LSNM modules (Figure 2).
3. We established new connections, from the connectome nodes that were originally connected to the TVB host node, to the “embedded” LSNM module (see Figure 3).
4. We also established feedback connections, from each one of the embedded LSNM submodules to the connectome nodes that were connected to a given submodule.

**Table 1:** **Hypothesized locations, in Talairach coordinates (Talairach and Tournoux, 1988), of visual LSNM modules, along with the closest node in the Hagmann et al. (2008) connectome**.

**Visual submodule**	**Talairach location**	**Source**	**Host connectome node**
V1/V2	(18, −88, 8)	Haxby et al., 1995	(14, −86, 7)
V4	(30, −72, −12)	Haxby et al., 1995	(33, −70, −7)
IT	(28, −36, −8)	Haxby et al., 1995	(31, −39, −6)
FS	Location selected for illustrative purposes		(47, 19, 9)
D1	(42, 26, 20)	Haxby et al., 1995	(43, 29, 21)
D2	Location selected for illustrative purposes		(42, 39, 2)
FR	Location selected for illustrative purposes		(29, 25, 40)

*Note that the locations of FS, D1, D2, and FR are not explicitly known (see text) and were chosen only to demonstrate validity of the method*.

Because each one of the embedded LSNM submodules greatly outnumber their TVB host node (e.g., by 81 to 1 in IT in the current paper), we divided the long-range coupling constant used in TVB among connectome nodes by the number of units contained within each submodule to be embedded (Figure 3). We used the result of this arithmetic division as a mean value to generate pseudo-random numbers with a Gaussian distribution, and used those numbers as a coupling term (details of which are provided below) from connectome nodes to LSNM units. Regarding the feedback connections from the embedded LSNM submodules to the TVB nodes, we used the long-range coupling constant provided by TVB to scale the connectome's structural connection weights (see details below).

The locations of the four PFC nodes (FS, D1, D2, FR) require some comment. As mentioned above, the inclusion of these four neural populations in the original LSNMs was based on the electrophysiological studies of Funahashi et al. (1990) that found in monkey PFC four distinct neuronal responses during a delayed response task: neurons that (1) increased their activity when a stimulus was present (represented in our model by FS), (2) increased their activity during the delay part of the task (D1), (3) increased their activity during both when a stimulus was present and during the delay period (D2), and (4) increased their activity prior to making a correct response (FR). It is not known if these neuronal types are found in separate anatomical locations in PFC or are intermixed within the same brain area, although the latter is the more likely case (except possibly for the FR population). In the original modeling studies of Tagamets and Horwitz (1998) and Husain et al. (2004), the functional neuroimaging data represented a single region that included all four nodes. To illustrate the integrated synaptic activity and fMRI signal for each one of the modules of the combined LSNM / TVB model separately, we have assigned a different spatial location to each one of the four PFC sub-modules. We have used the Talairach coordinates of the prefrontal cortex, based on Haxby et al. (1995), for the submodule D1 and have designated spatial locations in adjacent regions of interest for the rest of the submodules (see Table 1).

### Simulating electrical activity and fMRI activity

#### Electrical activities of each node in hagmann's connectome (TVB equations)

Each one of the nodes in Hagmann's connectome is represented as a Wilson-Cowan model of excitatory (E) and inhibitory (I) neuronal populations, as described in Sanz Leon et al. (2015):

$$\begin{array}{l} {\dot{E}}_{i}=\frac{1}{\tau_{E}}(\;-E_{i}\;+\;\left(k_{E}\;-\;r_{E}E_{i}\right)S_{E}[\alpha_{E}(c_{EE}E_{i}\;-\;c_{IE}I_{i}\;+\;-\theta_{E}\\ \;+\;\Gamma \left(E_{i},E_{k},u_{ik}\right))]) \end{array}$$

and

$$\begin{array}{l} {\dot{I}}_{i}=\frac{1}{\tau_{I}}(\;-I_{i}\;+\left(k_{I}-r_{I}I_{i}\right)S_{I}[\alpha_{I}(c_{EI}E_{i}\;-\;c_{II}I_{i}\;-\;\theta_{I}\\ \;+\;\Gamma \left(E_{i},E_{k},u_{ik}\right))]) \end{array}$$

where *S*_*E*_ and *S*_*I*_ are sigmoid functions described by

$$\begin{array}{l} S_{a}\left[f\left(\varphi \right)\right]=\frac{c}{1\;+\;e^{\left(-a\left(f\left(\varphi_{a}\right)-b\right)\right)}} \end{array}$$

*c*_*EE*_, *c*_*EI*_, *c_II_*, *c*_*IE*_ are the connections within the single neuronal unit itself; note that although the original TVB Wilson-Cowan population model allows us to consider the influence of a local neighborhood of neuronal populations, we have not used this feature in our current simulations and have left that term out of the equations above; **Γ**(*E*_*i*_, *E*_*k*_, *u*_*ik*_) is the long-range coupling function, defined as 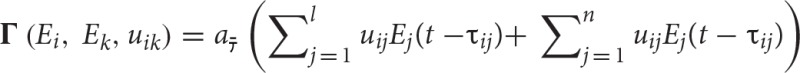 where *l* is the number of nodes in the connectome and *n* is the number of LSNM units connected to a connectome node; *a*_**Γ**_ is a constant that depends on the number of nodes in the connectome (see Tables S1, S2) for the definition and value of the parameters in the above equations).

#### Electrical activities of each LSNM unit

Each one of the submodules of the LSNM model contains 81 neuronal population units. Each one of those units is modeled as a modified Wilson-Cowan population of excitatory (*E*) and inhibitory (*I*) elements. The electrical activities of each one of those elements at time *t* is given by the following equations:

$$\begin{array}{lll} \frac{dE_{i}\left(t\right)}{dt} & = & △\left(\frac{1}{\;1+\;e^{\;-K_{E}\left[w_{EE}E_{i}\left(t\right)\;+\;w_{IE}I_{i}\left(t\right)\;+\;{in}_{iE}\left(t\right)\;-\;\phi_{E}\;+\;N\left(t\right)\right]}}\right)\\ -\;\delta E_{i}\left(t\right) \end{array}$$

and

$$\begin{array}{l} \frac{dI_{i}\left(t\right)}{dt}=△\left(\frac{1}{1\;+\;e^{\;-\;K_{I}\left[w_{EI}E_{i}\left(t\right)\;+\;{in}_{iI}\left(t\right)\;-\;\phi_{I}\;+\;N\left(t\right)\right]}}\right)\;-\;\delta I_{i}\left(t\right) \end{array}$$

where △ is the rate of change, δ is the rate of decay, *K*_*E*_, *K*_*I*_ are gain constants, ϕ_*E*_, ϕ_*I*_ are input threshold values, *N*(*t*) is a noise term, *w*_*EE*_, *w*_*IE*_, *w*_*EI*_ are the weights within a unit (the values of △, δ, *K*, τ, *N* are given in the Table S3); *in*_*iE*_(*t*), *in*_*iI*_(*t*) are the inputs coming from other brain regions at time *t*. *in*_*iE*_(*t*) is given by:

$$\begin{array}{l} {in}_{iE}\left(t\right)=\underset{j}{\sum }w_{ji}^{E}E_{j}\left(t\right)+\underset{j}{\sum }w_{ji}^{I}I_{j}\left(t\right)+\underset{j}{\sum }c_{ji}z_{ji}^{C}C_{j}\left(t\right) \end{array}$$

where $w_{ji}^{E}$ and $w_{ji}^{I}$ are the weights originating from excitatory (E) or inhibitory (I) unit *j* from another LSNM unit into the *i*th excitatory element, *C*_*j*_ is the connectome excitatory unit *j* with connections to the LSNM unit *i*, $z_{ji}^{C}$ is the value of the anatomical connection weight from connectome unit *j* to LSNM unit *i*, and *c*_*ji*_ is a coupling term, which was obtained by using Python's Gaussian pseudo-random number generator (*random.gauss*), using 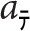 / 81 as the mean value. The input coming into the *i*th inhibitory element, *in*_*iI*_(*t*), is given by:

$$\begin{array}{l} {in}_{iI}\left(t\right)=\underset{k}{\sum }w_{ki}^{E}E_{k}\left(t\right)+\underset{k}{\sum }w_{ki}^{I}I_{k}\left(t\right) \end{array}$$

where $w_{ki}^{E}$ and $w_{ki}^{I}$ are the weights originating from excitatory (E) or inhibitory (I) unit *k* from another LSNM unit into the *i*th inhibitory element. Note that there are no connections from the connectome to LSNM inhibitory units. See Tables S4, S5 for details. Note also that, whereas TVB simulator incorporates explicit transmission delays among the connectome nodes, the LSNM nodes do not. The time step used in the LSNM model is 5 ms.

#### Integrated synaptic activity

Prior to computing fMRI BOLD activities we compute the synaptic activity, spatially integrated over each LSNM module (or connectome node) and temporally integrated over 50 ms as described by Horwitz and Tagamets (1999)

$$\begin{array}{l} rSYN=\underset{t,i}{\sum }{IN}_{i}\left(t\right) \end{array}$$

where *IN*_*i*_(*t*) is the sum of absolute values of all inputs to both *E* and *I* elements of unit *i*, at time *t*, and is given by:

$$\begin{array}{l} {IN}_{i}\left(t\right)=w_{EE}E_{i}\left(t\right)+\;w_{EI}E_{i}\left(t\right)+|w_{IE}I_{i}\left(t\right)|+\underset{k,i}{\sum }w_{ki}E_{k}\left(t\right) \end{array}$$

Note that the first three terms above are the synaptic weights from within unit *i* and the last term is the sum of synaptic connections originating in all other LSNM units and connectome nodes connected to unit *i*. Note that, in our current scheme, there are no long-range connections from inhibitory populations.

#### Generation of subjects and task performance of the LSNM model

We generated simulated subjects by creating a number of different sets of connection weights among submodules of the LSNM visual network until we obtained the number of desired subjects whose task performance accuracy was above 60%. The performance difference between subjects depends primarily on the amount of neural noise. The connection weights among the TVB connectome nodes remained unchanged across subjects. The generation of different connectome sets to simulate individual subjects is outside the scope of the current paper but will be essential for future simulation studies investigating the effects of a behavioral task on non-task brain nodes. Task performance was measured as the proportion of correct responses over an experiment. A response in the response module (FR, described in the caption to Figure 1) was considered a correct response in a given trial if at least 2 units had neuronal electrical responses above a threshold of 0.7 during the response period. To create different sets of weights that were different from the ideal subject, we multiplied feed forward connections among modules in the LSNM visual model by a random proportion of between 0.95 and 1.

#### Equations for the forward fMRI BOLD model

We implemented the BOLD signal model described by Stephan et al. (2007). We use the output of the integrated synaptic activity above as the neural state equation to the hemodynamic state equations below. The BOLD signal for each ROI, *y(t)*, is computed as follows:

$$\begin{array}{l} y\left(t\right)=V_{0}\left(k_{1}\left(1\;-\;q\right)\;+\;k_{2}\left(1\;-\;\frac{q}{v}\right)\;+\;k_{3}\left(1\;-\;v\right)\right) \end{array}$$

where the coefficients *k*_1_, *k*_2_, *k*_3_ are computed as:

$$\begin{array}{l} k_{1}\;=4.3\vartheta_{0}E_{0}TE\\ k_{2}\;=\varepsilon r_{0}E_{0}TE\\ k_{3}\;=1-\varepsilon \end{array}$$

where *V*_0_ is the resting venous blood volume fraction, *q* is the deoxyhemoglobin content, *v* is the venous blood volume, *E*_0_ is the oxygen extraction fraction at rest, ε is the ratio of intra- and extra-vascular signals, and *r*_0_ is the slope of the relation between the intravascular relaxation rate and oxygen saturation, ϑ_0_ is the frequency offset at the outer surface of the magnetized vessel for fully deoxygenated blood at 1.5T, and *TE* is the echo time. The evolution of the venous blood volume *v* and deoxyhemoglobin content *q* is given by the balloon model hemodynamic state equations, as follows:

$$\begin{array}{l} \tau_{0}\frac{dv}{dt}=f\left(t\right)-\;{v\left(t\right)}^{1/\alpha } \end{array}$$

$$\begin{array}{l} \tau_{0}\frac{dq}{dt}=f\left(t\right)\frac{1\;-\;{\left(1\;-\;E_{0}\right)}^{1/f}}{E_{0}}\;-\;{v\left(t\right)}^{1/\alpha }\frac{q\left(t\right)}{v\left(t\right)} \end{array}$$

where τ_0_ is the hemodynamics transit time, α represents the resistance of the venous balloon (vessel stiffness), and *f*(*t*) is the blood inflow at time *t* and is given by

$$\begin{array}{l} \frac{df}{dt}=s \end{array}$$

where *s* is an exponentially decaying vasodilatory signal given by

$$\begin{array}{l} \frac{ds}{dt}=\epsilon x-\;\frac{s}{\tau_{s}}\;-\;\frac{\left(f-1\right)}{\tau_{f}} \end{array}$$

where ϵ is the efficacy with which neuronal activity *x(t)* (i.e., integrated synaptic activity) causes an increase in signal, τ_*s*_ is the time constant for signal decay, and τ_*f*_ is the time constant for autoregulatory feedback from blood flow (Friston et al., 2000). For the values we use for all these parameters (Friston et al., 2000; Obata et al., 2004), see the Table S6. The resulting time series is downsampled to correspond to a TR value of 2 s.

## Results

In the previous section, we described a framework for inserting task-based neural network models into a connectome-based model of the cerebral cortex. Specifically, we embedded a LSNM of visual short-term memory into the connectome. The connectome acts as a source of noise, and therefore a source of variability, to the electrical activities of task-specific neuronal populations of the visual LSNM. The visual LSNM, in turn, incorporates extra connectivity into the connectome. Such extra connectivity is, as mentioned above, a refinement of the gross connectivity provided by the white-matter tract weights given by the connectome, and they are necessary for a computational model to perform a behavioral task. In order to embed the LSNM into the connectome, we selected specific spatial locations for each LSNM submodule.

**Figure 4:**
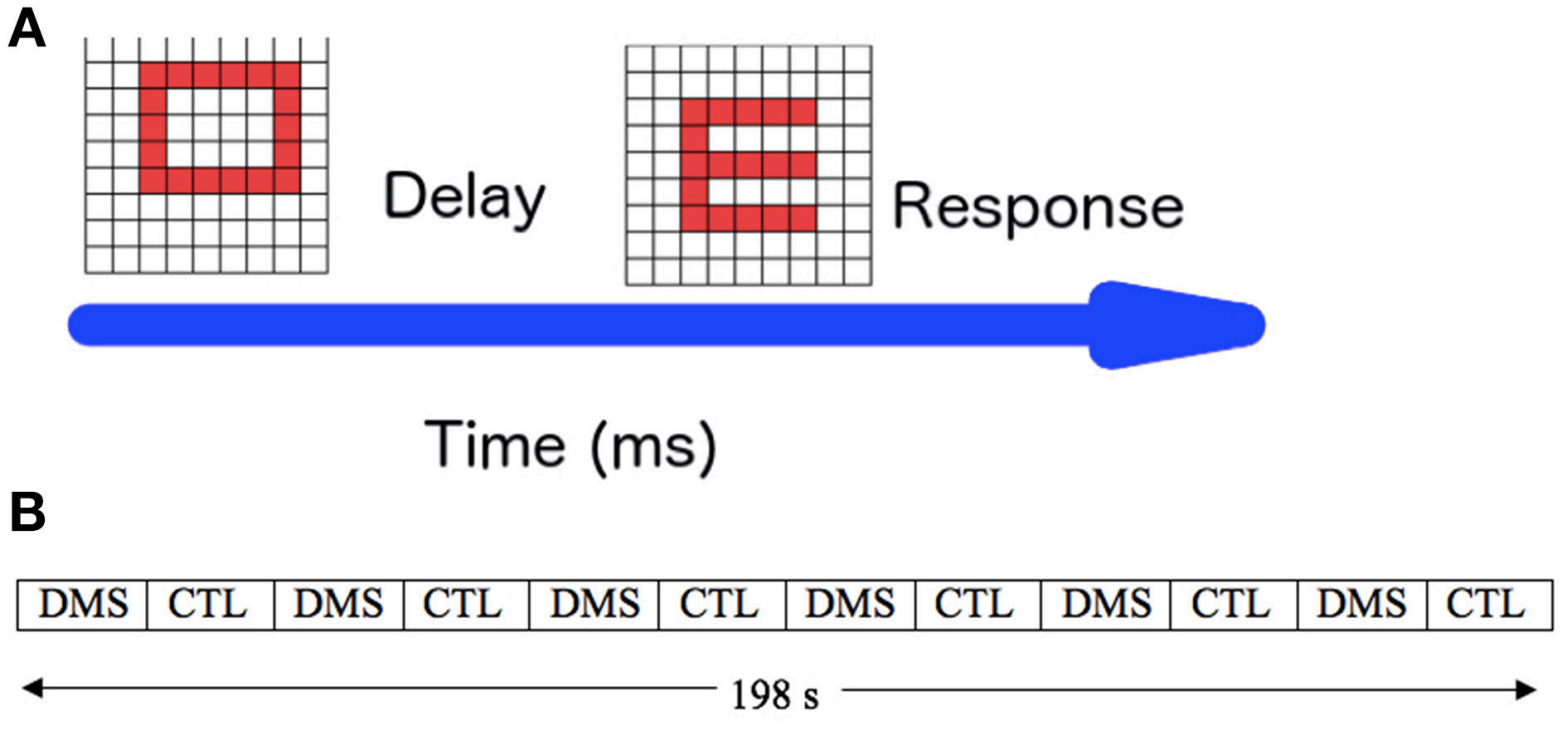
**Simulated experiment**. **(A)** One trial of the simulated visual delayed match-to-sample (DMS) task: a visual pattern S1 is presented for 1 s, followed by a 1.5-s delay period during which S1 has to be kept in short-term memory. When a second visual pattern (S2) is presented, the experimental subject has to respond whether S2 matches the memory of S1. **(B)** Timing of presentation of DMS and passive viewing (CTL) trials in a full experimental paradigm. Each block, DMS or CTL, consisted of 3 trials and was 16.5 s long. The total duration of the simulated experiment was 198 s.

To test our modeling framework, we used the standard visual DMS task described in the Overview section. To give our simulations as much realism as possible, we simulated an experiment consisting of multiple trials of the DMS task and passive viewing (control) task, as shown in Figure 4A. Our simulated experiment consisted of 36 trials, alternating three DMS trials and three control trials (Figure 4B). To facilitate a direct comparison of the current simulations with those of Horwitz et al. (2005), the attention level/task control parameter was varied during the DMS condition (range: 0.24–0.34). We simulated the passive viewing trials by using a constant low level of attention (0.05). Ten different subjects were generated by following the steps described in the Methods section.

### Electrical activity in a representative subject

**Figure 5:**
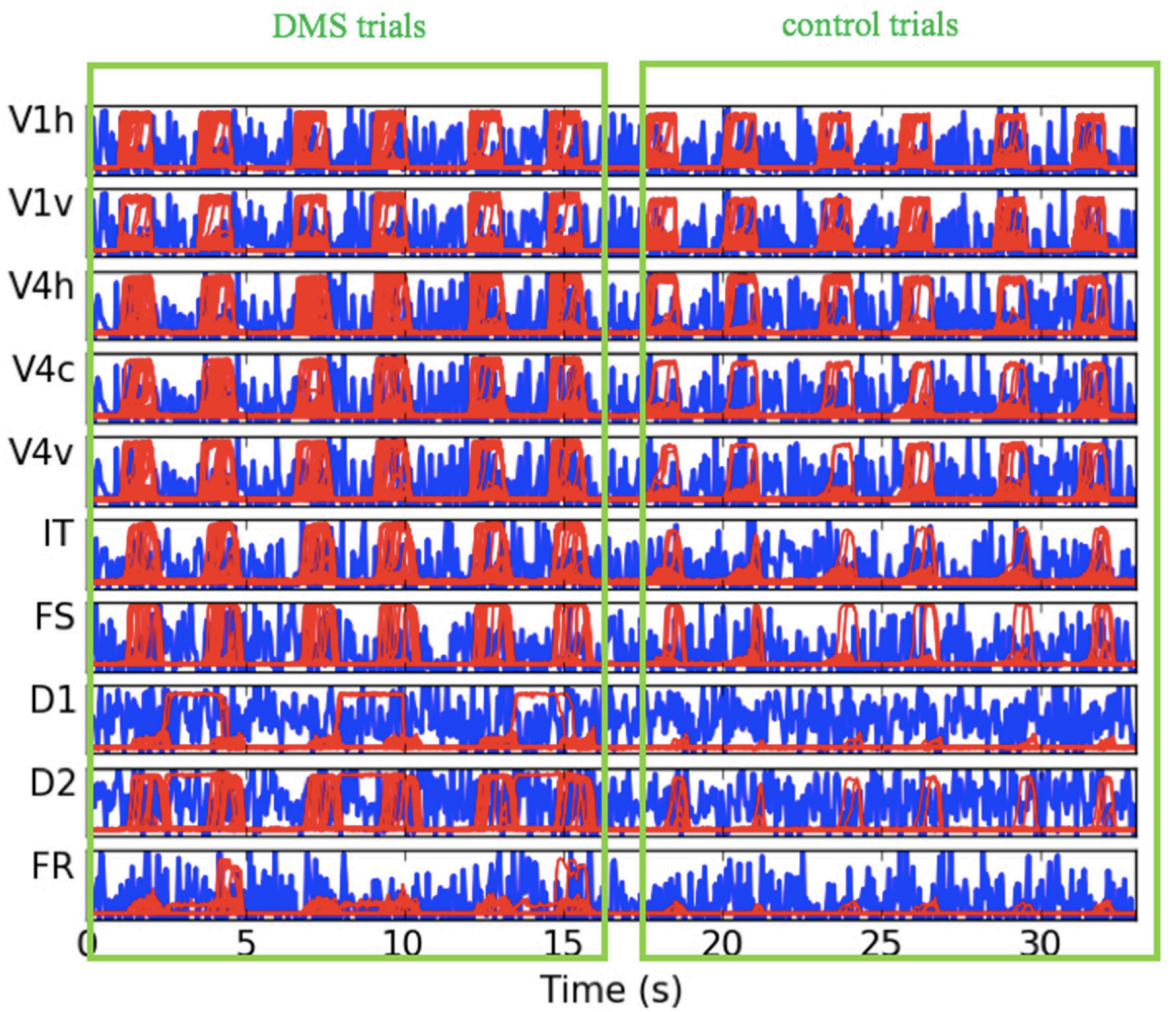
**Plots of electrical activity of all LSNM visual modules (red lines) and host nodes of Hagmann's connectome (blue lines), for a representative simulated subject**. The simulated tasks were: three DMS trials (match, mismatch, match), followed by three control trials where degraded shapes were used as inputs and the attention parameter was set to low (passive viewing). The y axis of each of the plots represents level of electrical activity, between 0 and 1. V1h and V1v denote vertical and horizontal-selective neuronal populations within V1/V2. V4h, V4c, and V4v denote vertical, corner, and horizontal-selective neuronal populations within V4.

The neural activity time-series for each one of the LSNM submodules and the respective connectome host nodes, for a representative subject, are shown in Figure 5. To be able to inspect the fine details of the simulated electrical activity, only the first six trials are shown (three DMS and three control). We are able to observe that the electrical activities during DMS trials as compared to control trials are of a similar strength in the V1 units and, to a lesser extent, in V4 LSNM submodules (red-line plots in Figure 5). However, if we look at the electrical activity in the IT and FS submodules (red-line plots in Figure 5), we observe that the electrical activity in IT and FS is stronger during the DMS trials than during the control trials. We also observe that D1 and D2 exhibit electrical activity during the delay period within DMS trials but not within passive viewing trials, and that D2's activity is stronger during stimulus presentation within DMS trials than during passive viewing trials. Finally, the electrical activity in FR shows a stronger response during the response period for a match (first and third DMS trial in Figure 5) than for a mismatch (second DMS trial in Figure 5). Additionally, during the control trials, the FR submodule exhibits a rather weak response for either match or mismatch. These results were expected and match the performance of the stand-alone LSNM visual short-term memory of (Tagamets and Horwitz, 1998; Horwitz et al., 2005); moreover, they are in agreement with the experimental findings of Funahashi et al. (1990). Regarding the difference between the DMS trials and the control trials in the IT, FS, D1, D2, and FR submodules, we know that the only difference between the DMS trials and the control trials is the different levels of attention/task control parameter used in those trials. Whereas we use a very low attention parameter for the control trials (0.05), we employed a higher level of attention for the DMS trials. As displayed in the LSNM visual model diagram shown in Figure 1A, the attention parameter directly modulates the electrical activity of D2 (as depicted by an arrow from “attention” to “D2”), and indirectly (through indirect connections through other submodules) affects the neural activity of V4, IT, FS, D1, and FR. Finally, note that the background noise shown by blue-line plots in Figure 5 represents the electrical activity of the connectome nodes that are acting as hosts to the respective LSNM submodules in the same plot; there appears to be no task-related difference in the activity pattern of the background noise units. The results shown in Figure 5 representing the combined LSNM-TVB model are quite similar to those shown in Figure 3 of Horwitz et al. (2005) for the LSNM stand-alone model, thus demonstrating a successful embedding of the LSNM into TVB.

### Integrated synaptic activity in a representative subject

**Figure 6:**
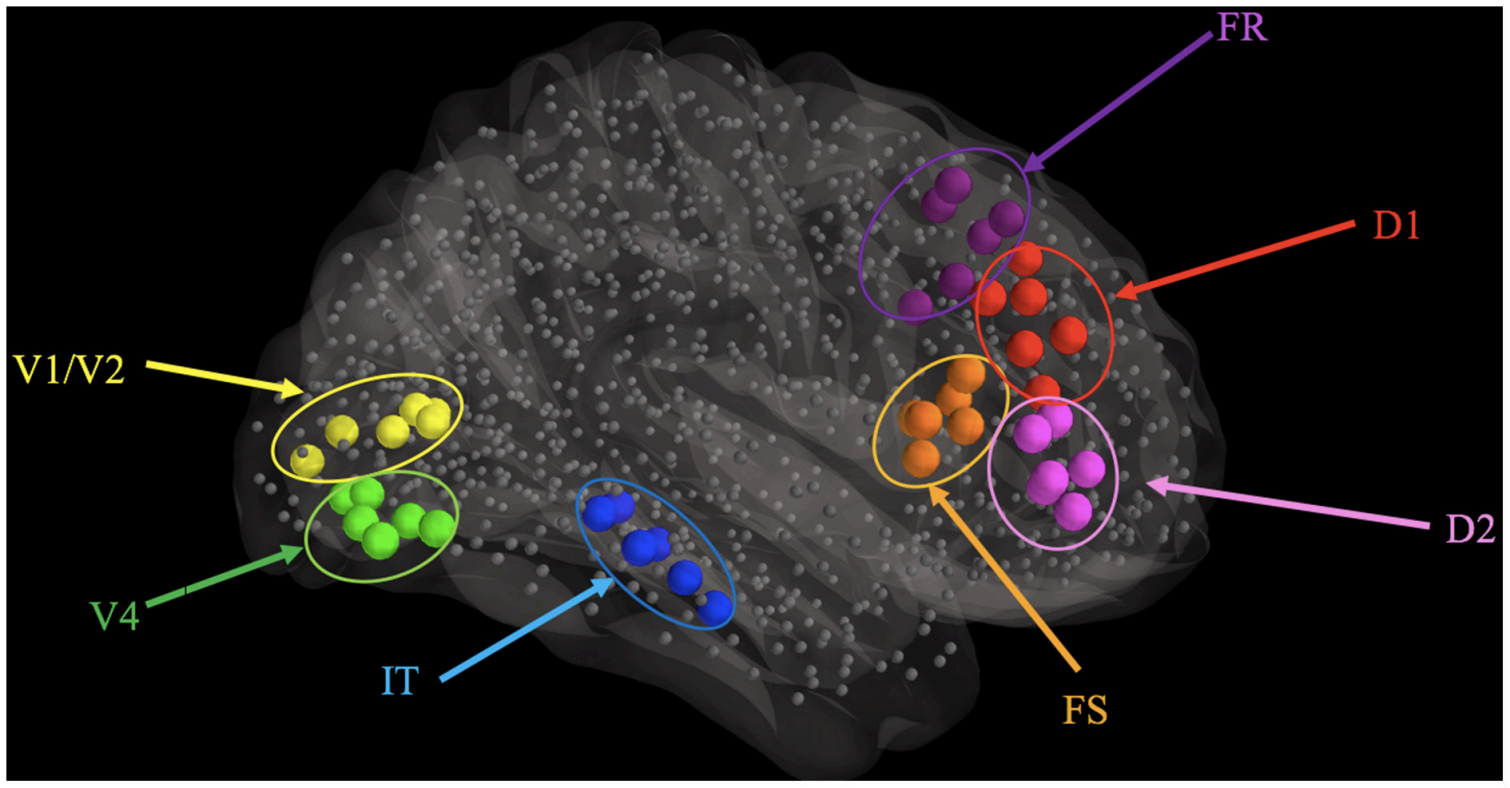
**Regions of interest used for computation of both integrated synaptic activities and fMRI BOLD time-series**.

**Figure 7:**
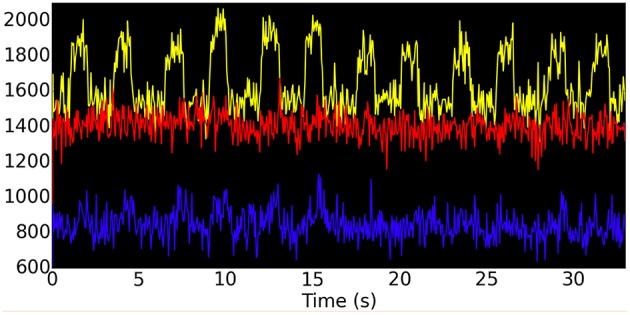
**Integrated synaptic activity using the ROIs for V1 (yellow), IT (blue), and D1 (red) shown in Figure 6 during a visual DMS experiment for a representative subject**. Only the first six trials of the experiment are shown, corresponding to the electrical activities shown in Figure 5. The y-axis is in arbitrary units and the x-axis represents time in seconds.

As a first step for computing the fMRI BOLD time-series for our representative subject, we computed the integrated synaptic activity (ISA) for select regions of interest (ROIs) in the hybrid LSNM/TVB model. These ROIs are composed of both task-related neuronal populations (our original LSNM submodules) and non-task-related neuronal populations (a number of TVB nodes spatially adjacent to the LSNM submodules that are not engaged in the task at hand). We arbitrarily selected five connectome nodes that were closest to each embedded LSNM submodule. Thus, our ROIs were composed of one embedded LSNM submodule, that includes the host connectome node, and five adjacent connectome nodes, as depicted in Figure 6. We then used the procedure outlined in the Methods section to compute the integrated synaptic activity of each ROI. Figure 7 shows the integrated synaptic activity for the ROIs corresponding to V1 (yellow), IT (blue), and D1 (red) during the first six trials for the same representative subject discussed earlier. This integrated synaptic activity is what we would see if we were able to image the synaptic activity in different brain regions directly; we will use it to compare with the analysis of the simulated BOLD time-series below. The y axis is in arbitrary units (and thus, no significance is attached to the magnitude differences between the different nodes) and the x axis represents time in seconds. Activity representing stimulus presentation is clearly evident in V1, but not in the other two ROIs.

### Simulated fMRI bold time-series

**Figure 8:**
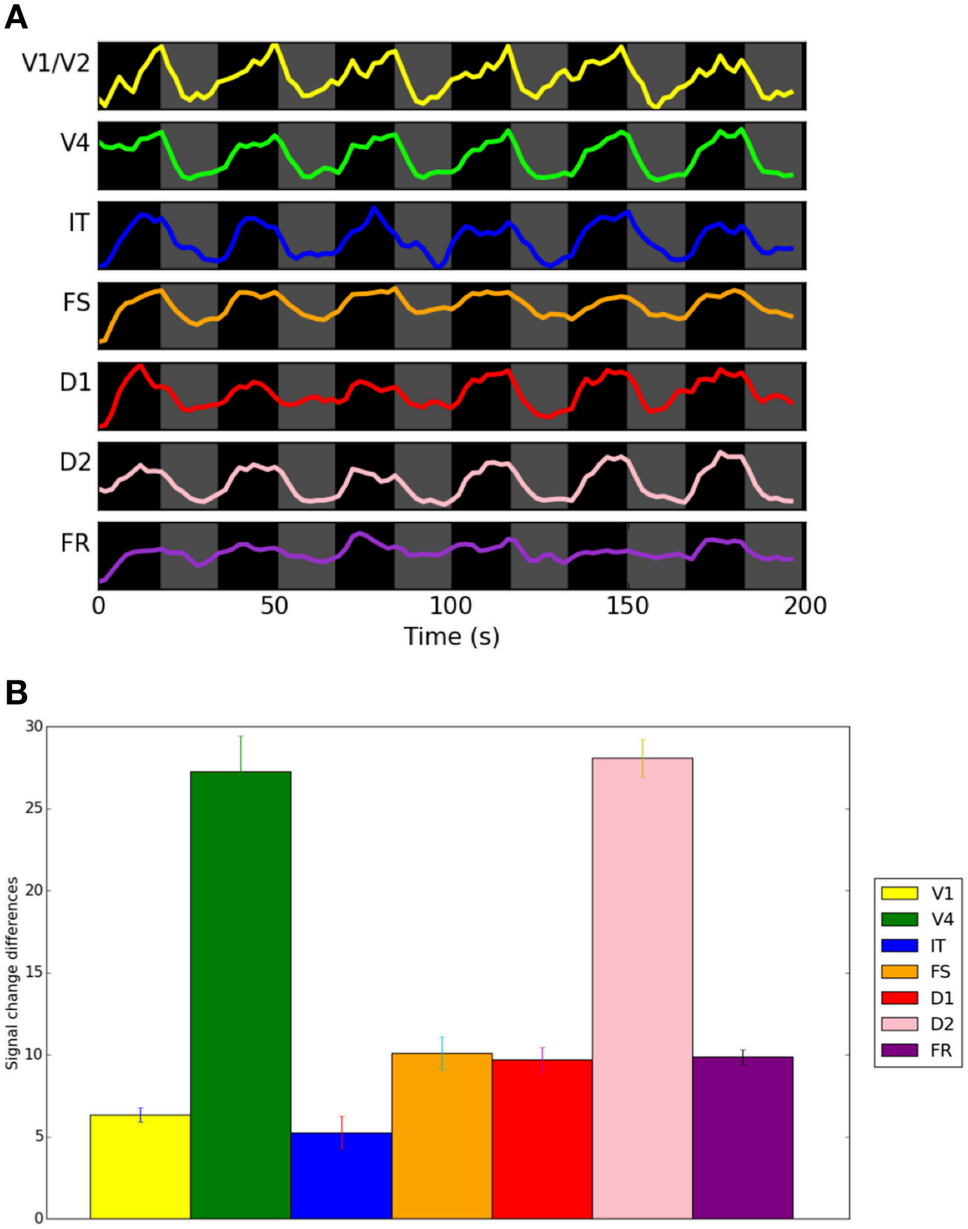
**(A)** Simulated fMRI BOLD signals, using the balloon hemodynamic response model, in combined LSNM/TVB modules, corresponding to the representative subject's integrated synaptic activity shown in Figure 7. Thirty-six trials were simulated, in groups of six task (DMS) trials (black areas) followed by six control (CTL) trials (gray areas). The x-axis represents time in seconds and the y-axis is in arbitrary units. The simulated TR is 2 s. **(B)** Bar graphs representing the mean signal change of timepoints 4, 5, and 6 after onset of a block of trials for each condition across 10 simulated subjects. In each ROI, the mean of the within-subject difference between the DMS and CTL conditions was statistically significant (see Table 3). Error bars indicate standard errors.

We used the integrated synaptic activity of each ROI as the input to the fMRI BOLD balloon model of hemodynamic response (see Methods section) to obtain a simulated fMRI signal time-series for each ROI using the same representative subject discussed above. Figure 8A shows these time-series during the length of the simulated experiment (198 s). By inspecting the time-series, we can appreciate, in some areas more clearly than others, that the simulated fMRI signal is greater during the DMS trials than during the control trials. Figure 8B shows the mean change in simulated fMRI across 10 subjects for the DMS task condition relative to the control condition. Performance data for these 10 simulated subjects is presented in Table 2; simulated fMRI was significantly higher for the DMS task compared to the passive viewing control condition (paired *t*-tests, *p* < 0.05 uncorrected; see Table 3).

**Table 2:** **Within subject average performances during the DMS condition, as measured by counting the number of neuronal populations in the response module (FR) responding above a certain threshold during the response period**.

**Subject**	**DMS Performance (%)**
S1	77.8
S2	72.2
S3	83.3
S4	66.7
S5	66.7
S6	61.1
S7	77.8
S8	83.3
S9	72.2
S10	77.8

**Table 3:** **Comparison of Signal Change of simulated fMRI across 10 simulated subjects during a visual DMS task vs. control task (passive viewing of degraded shapes)**.

	**DMS-mean**	**DMS-s.d**.	**Control-mean**	**Control-s.d**.	**Mean difference**	**t statistic**
V1/V2	2.5302	0.5472	−3.7968	0.8245	6.3269	14.4291^*^
V4	15.5375	3.0294	−11.7135	3.6004	27.2510	12.4791^*^
IT	3.0592	1.3521	−2.2049	1.6807	5.2641	5.3149^*^
FS	6.7389	1.3770	−3.3634	1.7132	10.1023	10.1016^*^
D1	7.8545	1.2092	−1.8398	1.0691	9.6943	12.8041^*^
D2	17.2893	1.7016	−10.7798	1.8173	28.0691	24.0932^*^
FR	6.5407	0.7699	−3.3149	0.5918	9.8556	21.9574^*^

*The significance test used was a paired t-test with degrees of freedom = 9*.

* *p < 0.05 (uncorrected)*.

### Functional connectivity between brain regions

**Figure 9:**
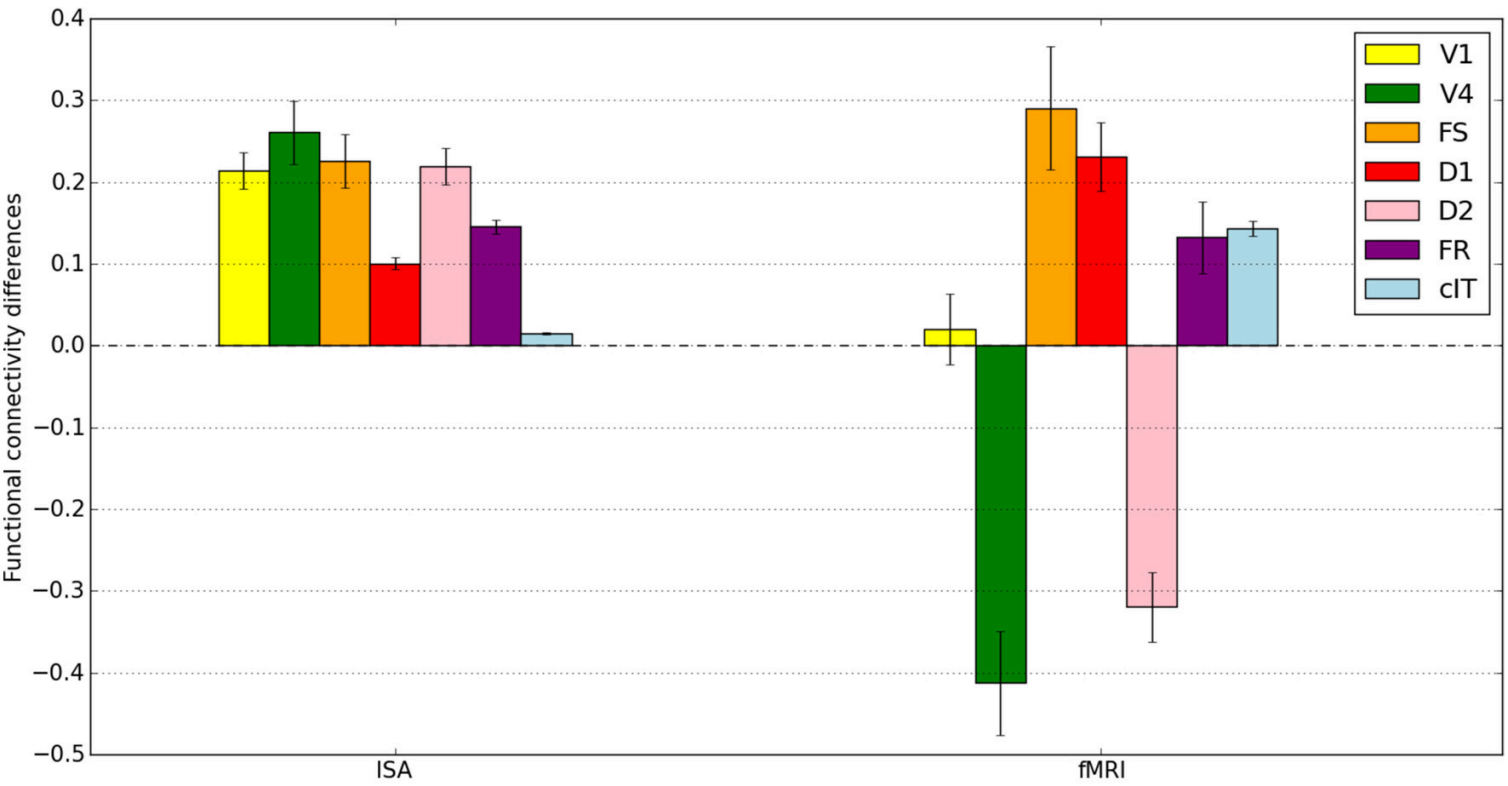
**Mean across subjects of the within-subject functional connectivity task differences between IT and all other brain modules**. Shown on the left are the functional connectivity task differences for the integrated synaptic activity (ISA) and on the right for the fMRI BOLD time-series. The differences between DMS and CTL in the ISA time-series all reached statistical significance; all the differences (except for V1) between DMS and CTL in the fMRI time-series also were statistically significance (see Table 4 for more details). Error bars represent standard errors.

To further compare our hybrid LSNM/connectome model's performance with the stand-alone model presented in Horwitz et al. (2005), we averaged functional connectivity values (defined as the within-condition correlation coefficient of the time series between two regions) across subjects (applying a Fisher Z transformation prior to averaging). Figure 9 shows the mean of the within-subject functional connectivity task difference between IT and each simulated ROI using both the integrated synaptic activity time-series and the fMRI BOLD signal time-series, and Table 4 presents the results of a statistical comparison between the two conditions. For comparison purposes, we also show in Figure 9 the functional connectivity of IT with the contralateral IT (which should have a low value, since activity in the contralateral IT consists entirely of noise). Figure 9 and Table 4 demonstrate that there is a much stronger functional connectivity, expressed as a within-task time-series correlation coefficient, between the ISA in IT and all other brain regions (except cIT), during the DMS task as compared to the control task. This result is what one would expect, knowing the network architecture.

**Table 4:** **Task differences in functional connectivities of IT and all other brain areas across 10 simulated subjects between a visual DMS task and control task (passive viewing of degraded shapes) for both the simulated integrated synaptic activities (ISA) and simulated fMRI BOLD**.

	**DMS mean**	**DMS s.d**.	**Control mean**	**Control s.d**.	**Mean difference**	**t statistic**
**ISA**						
V1	0.4315	0.0415	0.2171	0.0611	0.2144	9.7492^*^
V4	0.5482	0.0671	0.2872	0.0981	0.2609	6.7897^*^
FS	0.3396	0.0626	0.1134	0.0782	0.2262	6.8929^*^
D1	0.1384	0.0101	0.0379	0.0190	0.1005	13.5152^*^
D2	0.2874	0.0413	0.0675	0.0529	0.2199	9.8959^*^
FR	0.1523	0.0172	0.0067	0.0178	0.1456	17.2853^*^
cIT	−0.0022	0.0019	−0.0173	0.0028	0.0151	12.0941^*^
**fMRI**						
V1	0.8265	0.0991	0.8065	0.1796	0.0201	0.4653
V4	0.6477	0.1488	1.0601	0.2323	−0.4124	−6.5015^*^
FS	1.0479	0.0758	0.7572	0.2340	0.2906	3.8488^*^
D1	1.0075	0.1727	0.7761	0.1649	0.2315	5.4840^*^
D2	0.7739	0.1649	1.0936	0.2014	−0.3197	−7.5872^*^
FR	0.7640	0.1164	0.6312	0.1464	0.1328	3.0321^*^
cIT	0.2162	0.0406	0.0729	0.0453	0.1434	15.4369^*^

*The significance test used was a paired two-tailed t-test with degrees of freedom = 9*.

* *p < 0.05 (uncorrected)*.

Regarding the functional connectivity between the BOLD time-series of IT versus the BOLD time-series of all other ROIs (also shown in Figure 9), our results are mixed. We observe that, whereas the mean BOLD functional connectivity difference values between IT and FS, D1, and FR are statistically greater during the DMS condition than during the control condition (as expected), the functional connectivity values between IT and V4 and D2 are smaller during the DMS condition than during the control condition. Thus, the functional connectivity values of the fMRI BOLD time-series do not fully reflect the functional connectivity values obtained using the integrated synaptic activity time-series. This is not surprising since the ISA has a finer temporal resolution than does the fMRI, and futhermore, the hemodynamic response blurs together various aspects of the neural processing. This is similar to what was found in Horwitz et al. (2005).

### Stimulation results using only the connectome

**Figure 10:**
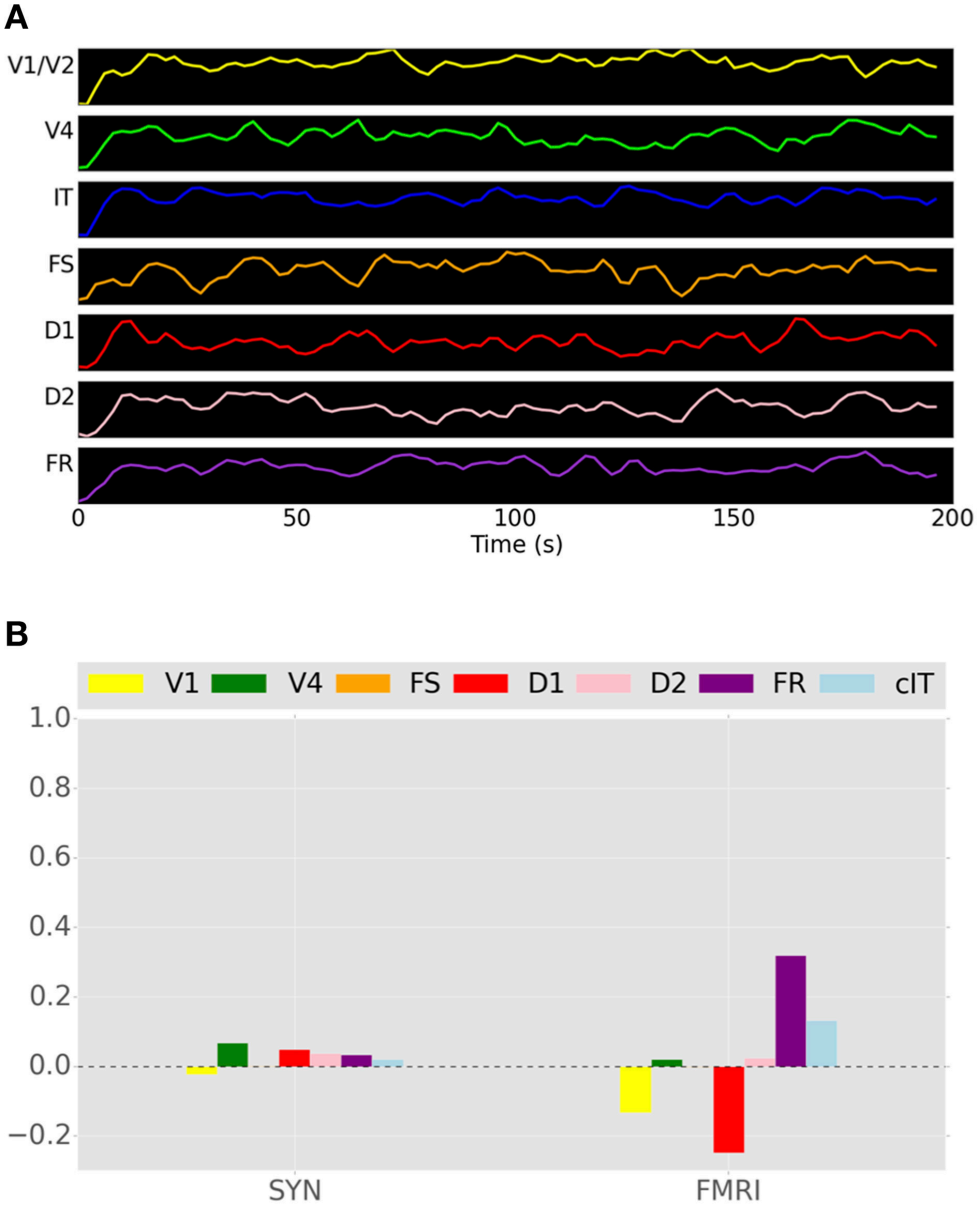
**(A)** Simulated BOLD signal of the unmodified TVB Hagmann et al. (2008) brain model during visual stimulation in a single subject. One of the nodes in V1 was stimulated with a series of pulse trains that were similar in timing to the stimuli presented during the DMS task. **(B)** Functional connectivity between IT and all other ROIs in the network using both integrated synaptic activities and fMRI BOLD time-series.

The question may arise: is the addition of our LSNM adding anything new? That is, would the TVB, as originally constructed, be able to perform a visual processing task and show the appropriate simulated electrophysiological activities? To compare the functional connectivity values of the hybrid LSNM/TVB model vs. TVB alone, we simulated a “V1 stimulation” paradigm using TVB. We created a stimulus pattern that was similar in structure to the input stimuli that was used in the hybrid LSNM/TVB simulations described above. The connectome node in which the V1 LSNM was to be embedded was stimulated with a train of 1-s pulses, 1.5-s delay period, and 2-s inter-trial intervals. The timing of stimulation was the same as the timing of stimulus presentation shown in Figure 4A. The duration of the stimulation experiment was 198 s and only one subject was simulated. Because we did not have an attention node in TVB, we did not simulate a control condition, and therefore all trials were, in fact, analogous to the passive viewing condition used in the hybrid LSNM/TVB model. Figure 10A shows the simulated BOLD time-series for that experiment, and Figure 10B shows the functional connectivity between the IT ROI and all other ROIs. We are able to observe in Figure 10B a set of very small positive correlation coefficients using the ISAs and a set of both positive and negative correlation coefficients using the fMRI time-series. The patterns of functional connectivity for both the ISA and fMRI look nothing like those seen in Figure 9. This result is as expected because in this case, essentially neural noise is the only thing being transmitted through the network. Thus, neither the simulated fMRI activities nor the functional connectivities are similar to those found for the simulations incorporating the task-based LSNM. These results demonstrate that the DSI-based structural connection weights are incapable of yielding the patterns of visual processing activity found in the task-based LSNM.

## Discussion

In this paper, we demonstrated how to embed a previously or newly constructed LSNM that performs one or more specific cognitive tasks (in our case, a visual DMS for object shape and a passive viewing of objects) into a structural connectome model of the human cerebral cortex that is part of TVB software framework. In work that will be presented elsewhere, we also accomplished this for a LSNM of the auditory object processing pathway that performs an auditory object DMS task (Horwitz et al., in preparation). The final result of the current paper is a full cerebral cortex model that performs both a visual DMS task as well as a control passive viewing task in a part of the brain, and generates inherent activity in the remaining parts of the brain not directly engaged by the task. Neural noise generated by the latter does affect the task-related nodes, which in turn have feedback connections back to the non-task related nodes. Importantly, this hybrid model can perform the cognitive tasks because the task-based nodes are connected by a more finely detailed set of anatomical connections than provided by the white matter DSI connection weights found in the structural connectome model supplied by the TVB software.

The benefits of the hybrid LSNM/TVB framework proposed in this paper are threefold. First, our framework adds a biologically plausible source of neural noise—originating from the TVB connectome nodes—to the electrical activities of task-specific neuronal populations (the LSNM nodes). Previous modeling work (Horwitz et al., 2005) had implemented neural noise by adding *ad-hoc* model regions that provided noise to task-specific neurons, but the new approach allows the addition of generic connectome data sets that have been obtained independently from the modeling work. Second, our framework incorporates extra connectivity into the TVB connectome. Such extra connectivity is a refinement of the gross connectivity provided by the white-matter tract weights given by the TVB connectome, and they are necessary for a model to perform a behavioral task. Previous computational models of the brain using the connectome have simulated resting-state but not task-based experiments. Finally, our framework compels modelers to select specific spatial locations within the connectome for putative task-based neuronal populations. Many previous modeling approaches did not have the constraint of having to specify explicit locations within the brain for model elements.

Most of the simulations we presented here were designed to show that they yielded results that were in agreement with those produced by the original LSNM (Tagamets and Horwitz, 1998; Horwitz and Tagamets, 1999; Horwitz et al., 2005), which themselves matched empirical electrophysiological data from non-human primates and functional neuroimaging (e.g., fMRI) data from human subjects. Because the simulated data from the original LSNM generally agreed with empirical findings, the combined model we have constructed shows comparable computational-experimental agreement. Thus, this work presents a template for expanding the TVB from a resting state modeling software package into a task-based framework.

Over the years, a large number of task-based neural models have been published, some of which have simulated human and non-human primate functional neuroimaging data. As we have repeatedly emphasized (e.g., Horwitz et al., 1999; Horwitz and Banerjee, 2012), functional neuroimaging data, being non-invasive, provide a major source of brain-based data from healthy humans as well as from many individuals with various types of brain disorders, and thus play a central role in our attempts to understand the neural basis of cognitive function (and its dysfunction), especially for those cognitive functions such as language that are uniquely human. Examples of this literature from other research groups include (Corchs and Deco, 2002; Deco et al., 2004; David et al., 2005; Robinson et al., 2005; Goebel and De Weerd, 2009; Peters et al., 2010; Bojak et al., 2011; Furtinger et al., 2014). Several other large-scale neural modeling efforts, although not specifically directed at functional neuroimaging data, nonetheless could be easily extended to simulate such data (e.g., Garagnani et al., 2008; Eliasmith et al., 2012; Garagnani and Pulvermuller, 2013).

Biologically realistic LSNM serve two functions. First, and certainly most important, such models embody neural mechanisms hypothesized to implement the specific cognitive tasks under investigation. For example, in the Tagamets-Horwitz LSNM (Tagamets and Horwitz, 1998) used in this paper, the way in which a representation is maintained in short-term memory during the delay portion of the DMS task is hypothesized to depend on the wiring pattern of four PFC neural populations. In the paper by Peters et al. (2010), a LSNM is used to simulate early visual processing of brightness changes in a dynamic, illusory display. The key hypothesis of their model, which is supported by anatomical and neurophysiological evidence, is that there are separate but interacting streams of processing related to the processing of contour boundaries and the processing of surfaces in early visual cortex.

The second function that LSNMs support is to provide partial validation for the interpretations arising from novel functional neuroimaging analysis methods. Unlike real brains, a LSNM can provide a ground truth for assessing a data analysis method, since every aspect of the model is known. For instance, the Tagamets and Horwitz (1998) visual processing model was used by Lee et al. (2006) to generate simulated fMRI data that could be analyzed by Dynamic Causal Modeling (DCM) (Friston et al., 2003); it was found that DCM produced strong evidence for those causal models with correctly specified anatomical connectivity corresponding to the underlying neural model. In another study, Banerjee et al. (2012a) employed the same visual model and generated simulated MEG data that were analyzed using a method that compares timing differences during network performance between two distinct tasks (Banerjee et al., 2012b). The simulated results supported the interpretation that the data analysis method would have drawn concerning the underlying neural network behavior mediating the tasks.

It is important to emphasize that connectome models obtained from diffusion tensor imaging data, such as employed by the TVB, are inadequate to explain the specific neural basis underlying any particular cognitive function (e.g., Figure 10). One reason is that structural connectivity data acquired from diffusion tensor/spectrum imaging lead to symmetric connection weights, since the direction of the white matter fibers between nodes cannot be determined by this technique. Usually for task-based models, the connection weights between nodes are asymmetric. A second reason is that the interregional connectivity weights derived from DTI are simply too crude. Task-based models, such as those mentioned above and those we employed in this paper, have a much finer and detailed set of interregional connection weights. It may be that advances in human structure imaging will result in more refined DTI-like measurements in the future, but such measurements will still likely be at a spatial scale that is too large to yield a set of weights that can enable a neural model to perform a specific task.

An approach somewhat similar to the one presented in this paper was developed by Goebel and colleagues (Goebel and De Weerd, 2009; Peters et al., 2010, 2012). The key notion was that one should combine task-based neural modeling within a whole-brain framework, so that functional neural imaging and neural modeling data can be directly compared. Specifically, Goebel and colleagues developed what was called a “common brain space” framework in which the neural elements of a computational model are connected to vertices of a cortical mesh in such a way as to implement specific hypotheses about how a task is mediated. In this common brain space, both simulated computational data and experimental functional neural imaging topographic data could be explicitly compared using exactly the same data analytic tools. Conceptually, the notion presented in this paper is similar, with the exception that instead of a cortical mesh, we employ the TVB connectome nodes. Our approach entails a larger cortical network, while the Goebel et al. framework has produced results at a finer spatial scale, specifically one that can be related to visual system neuroimaging data.

In the past, when a large-scale neural network model was devised, one could assign spatial names to the network modules without worrying about the actual topographic location of the computational modules. However, in the framework proposed here, these modules had to be placed into an actual cerebral cortical connectome each of whose nodes had specific spatial coordinates. There are likely to be a number of ways to decide which neural modeling node corresponds to which connectome node. In this paper, we used Talairach coordinates (Talairach and Tournoux, 1988) obtained from experimental task data (Haxby et al., 1995) for assigning topographic locations to the computational modules. The one area where this method did not work was in the PFC. As mentioned above, four distinct neural populations formed the PFC module, but it was not known if all four should be in the same macroscopic spatial location or in different locations. This is an important topic future research will need to address, since we know from experiments in non-human primates that a brain area is likely to be comprised of multiple neural populations (i.e., a single fMRI voxel contains neurons from several cortical columns and several cortical layers). Ultrahigh field fMRI is currently providing data that can somewhat resolve cortical columns or layers (for a review, see Bandettini et al., 2012).

A related issue is how many TVB nodes to include in any functional brain imaging ROI. In previous work (Horwitz et al., 2005), we indicated that a brain imaging ROI contained neural elements that participated in the task of interest, and other neural elements (non-specific nodes) that did not. However, because of the low spatial resolution of functional neuroimaging data, the neural activity of these latter elements would contribute to the measured neuroimaging data. In the combined model presented in this paper, we placed an arbitrary number (i.e., 5) of non-specific connectome nodes into each ROI of the task-based modules. Because these non-specific nodes are processing only noise, their contribution to the simulated fMRI signal is small. Nonetheless, future research will need to address the question of how to determine the appropriate number of such non-specific elements.

In the LSNM used in the current paper, a number of simplifications were employed that may have to be modified in future studies where more detailed neural models are employed. For example, in the current paper, we assumed there were no conduction delays between the various neural populations. This may be justified here by the fact that we didn't employ spiking neurons, used a task whose temporal resolution was in the range of seconds, and targeted fMRI where the BOLD signal is delayed by 4–6 s. Simulating EEG/MEG could necessitate, depending on the specific task that is under study, the incorporation of conduction delays into the model. For instance, both Ghosh et al. (2008) and Deco et al. (2009) found that important questions about resting state fMRI required employing neural models that include such delays. Note also that the Wilson-Cowan units used in the TVB differed somewhat from the LSNM modified Wilson-Cowan units. Future studies should clarify how these two sets differ with respect to network dynamics.

The connectome provides an anatomical starting point for extending the model so that it can perform other tasks, as well as the original one. The strength of the DSI anatomical connection weights provided by the connectome offer a useful hint as to where to insert the new modules that would extend the model's capability. This we see as a positive feature of the task-based connectome framework that is being proposed.

Finally, one more future research issue that our approach generates is to assess the effect of the task-based elements on the non-task based connectome nodes. Recall that there are bidirectional connections between the two sets of nodes. The non-task based nodes provide neural noise to the task-based elements, but the activity of the task-based elements in turn project back to the non-task based nodes. Two questions arise that could be compared with experimental data. First, how does the task-based activity affect the activity of the rest of the brain? Does it change its resting state character in any way? Second, will the task-based activity of one node affect others in the task-based part of the brain via activity it sends out to the non-task nodes which returns as noise to other task-based elements?

In conclusion, we have demonstrated how to embed a large-scale, biologically realistic task-based neural model into TVB, which provides a detailed connectome of the human cerebral cortex, neural processing units for each node of the connectome, and a set of forward models that can convert the simulated neural activity into a variety of functional brain imaging signals. Such a system will enable a better comparison between empirical and computational data, and lead to a better understanding of how interacting neural populations can lead to high level human cognitive behaviors.

## Author contributions

AU, BH designed the study; AU constructed the programs used and performed the simulations; AU, BH analyzed the simulated data. AU, BH drafted the manuscript.

### Conflict of interest statement

The authors declare that the research was conducted in the absence of any commercial or financial relationships that could be construed as a potential conflict of interest.
