# Supplementary material for: Embedding Task-Based Neural Models into a Connectome-Based Model of the Cerebral Cortex
Source: Front Neuroinform. 2016 Aug 3;10:32. doi: 10.3389/fninf.2016.00032 (PMC4971081; doi:10.3389/fninf.2016.00032)
Supplement: Supplementary file 1 [file Table1.PDF]

Table S1. Parameters used in the Wilson-Cowan equation for each connectome node within TVB. The parameters shown above are the default parameters within TVB and are also shown in Table 11(a) of Sanz Leon et al. (2015).

Parameter	Description	Value
c_{EE}	Excitatory to excitatory weight	12.0
c_{IE}	Inhibitory to excitatory weight	4.0
c_{EI}	Excitatory to inhibitory weight	13.0
c_{II}	Inhibitory to inhibitory weight	11.0
τ_E	Membrane time-constant, excitatory population	10.0
τ_I	Membrane time-constant, inhibitory population	10.0
a_E	Slope of excitatory response function	1.2
b_E	Position of maximum slope of excitatory sigmoid function	2.8
c_E	Amplitude of excitatory response function	1.0
θ_E	Excitatory threshold	0.0
a_I	Slope of inhibitory response function	1.0
b_I	Position of maximum slope of inhibitory sigmoid function	4.0
θ_I	Inhibitory threshold	0.0
c_I	Amplitude of inhibitory response function	1.0
r_E	Excitatory refractory period	1.0
r_I	Inhibitory refractor period	1.0
k_E	Maximum value of excitatory response function	1.0
k_I	Maximum value of inhibitory response function	1.0
α_E	Balance between excitatory and inhibitory	1.0
α_I	Balance between excitatory and inhibitory	1.0
