# Supplementary material for: Embedding Task-Based Neural Models into a Connectome-Based Model of the Cerebral Cortex
Source: Front Neuroinform. 2016 Aug 3;10:32. doi: 10.3389/fninf.2016.00032 (PMC4971081; doi:10.3389/fninf.2016.00032)
Supplement: Supplementary file 2 [file Table2.PDF]

Table S2. Parameters used for simulating the Hagmann et al. (2008) connectome within the TVB resting state simulator

Parameter	Value
**Number of nodes**	998
**Global coupling strength**	0.0042
**White matter transmission speed (mm/ms)**	4.0
**Integrator**	Euler stochastic (dt=5)

.
