# Supplementary material for: Embedding Task-Based Neural Models into a Connectome-Based Model of the Cerebral Cortex
Source: Front Neuroinform. 2016 Aug 3;10:32. doi: 10.3389/fninf.2016.00032 (PMC4971081; doi:10.3389/fninf.2016.00032)
Supplement: Supplementary file 3 [file Table3.PDF]

Table S3. Parameters used in the Wilson-Cowan unit model of each LSNM submodule

Parameter	E element	I element
K	9.0	20.0
$\boldsymbol{\phi}$	0.3	0.1
N	± 0.025	± 0.025
Δ	0.5	0.5
$\boldsymbol{\delta}$	0.5	0.5
