# Supplementary material for: Embedding Task-Based Neural Models into a Connectome-Based Model of the Cerebral Cortex
Source: Front Neuroinform. 2016 Aug 3;10:32. doi: 10.3389/fninf.2016.00032 (PMC4971081; doi:10.3389/fninf.2016.00032)
Supplement: Supplementary file 4 [file Table4.PDF]

Table S4. Connection patterns among submodules of LSNM model

Source	Destination	Fanout	Mean/SD	Percent to create	Comments
LGN	V1	7x7	34 @ 0.003±0.003<br>2 x 5 @ 0.006 ± 0.003<br>1 x 5 @ 0.020 ± 0.002	100	Highest values oriented either vertically or horizontally
V1h	V4h	1x5	0.04 ± 0.01	50	
V1v	V4v	5x1	0.04 ± 0.01	50	
V1h	V4c	3x3	4 @ 0.0 ± 0.01<br>5 @ 0.02 ± 0.01	50	Lowest values at the corners
V1v	V4c	3x3	4 @ 0.0 ± 0.01<br>5 @ 0.02 ± 0.01	50	Lowest values at the corners
V4	IT	5x5	0.01 ± 0.01	50	Learned
IT	FS	1x1	0.2 ± 0.02	100	
D2	V4	5x5	0.0014 ± 0.0007	100	
D1	IT	1x1	0.03 ± 0.001	100	Inhibitory
D2	IT	1x1	0.01 ± 0.002	100	
IT	V4	4x4	0.00125 ± 0.0006	100	
