# Supplementary material for: Embedding Task-Based Neural Models into a Connectome-Based Model of the Cerebral Cortex
Source: Front Neuroinform. 2016 Aug 3;10:32. doi: 10.3389/fninf.2016.00032 (PMC4971081; doi:10.3389/fninf.2016.00032)
Supplement: Supplementary file 5 [file Table5.PDF]

Table S5. Connection weights among submodules in the prefrontal cortex region of LSNM

Source	Destination	Element	Weight
FS	D2	E	0.07
FS	FR	E	0.05
D1	FR	E	0.06
D1	D2	E	0.105
D2	D1	E	0.10
D1	FS	I	0.02
FS	D1	I	0.05
FR	D1	I	0.03
FR	D2	I	0.065
