# Supplementary material for: Embedding Task-Based Neural Models into a Connectome-Based Model of the Cerebral Cortex
Source: Front Neuroinform. 2016 Aug 3;10:32. doi: 10.3389/fninf.2016.00032 (PMC4971081; doi:10.3389/fninf.2016.00032)
Supplement: Supplementary file 6 [file Table6.PDF]

Table S6. Parameters used for the Balloon model of hemodynamic response used in our simulations. Values are based on Friston et al. (2000) and Obata et al. (2004).

Parameter	Description	Value
$\boldsymbol{\tau_s}$	Rate constant of vasodilatory signal decay in seconds	1.5
$\boldsymbol{\tau_f}$	Time of flow-dependent elimination in seconds	4.5
$\boldsymbol{\alpha}$	Grubb's vessel stiffness exponent	0.2
$\boldsymbol{\tau_0}$	Hemodynamic transit time in seconds	1.0
$\boldsymbol{\epsilon}$	Efficacy of synaptic activity to induce signal	0.1
$\boldsymbol{r_0}$	Slope of intravascular relaxation rate in Hertz	25.0
$\boldsymbol{\vartheta_0}$	Frequency offset at outer surface of magnetized vessels	40.3
$\boldsymbol{\varepsilon}$	Ratio of intra- and extravascular BOLD signal at rest	1.43
$\boldsymbol{V_0}$	Resting blood volume fraction	0.02
$\boldsymbol{E_0}$	Resting oxygen extraction fraction	0.8
$\boldsymbol{TE}$	Echo time, 1.5T scanner	0.04
